# Ferroptosis and EMT: key targets for combating cancer progression and therapy resistance

**DOI:** 10.1007/s00018-023-04907-4

**Published:** 2023-08-19

**Authors:** Yuqing Ren, Xiangrong Mao, Hui Xu, Qin Dang, Siyuan Weng, Yuyuan Zhang, Shuang Chen, Shutong Liu, Yuhao Ba, Zhaokai Zhou, Xinwei Han, Zaoqu Liu, Guojun Zhang

**Affiliations:** 1 Department of Respiratory and Critical Care Medicine, The First Affiliated Hospital of Zhengzhou University, Zhengzhou, 450052 Henan China; 2 Department of Ophthalmology, The First Affiliated Hospital of Zhengzhou University, Zhengzhou, 450052 Henan China; 3 Department of Interventional Radiology, The First Affiliated Hospital of Zhengzhou University, Zhengzhou, 450052 Henan China; 4 Department of Colorectal Surgery, The First Affiliated Hospital of Zhengzhou University, Zhengzhou, 450052 Henan China; 5 Center of Reproductive Medicine, The First Affiliated Hospital of Zhengzhou University, Zhengzhou, Henan China; 6 Department of Pediatric Urology, The First Affiliated Hospital of Zhengzhou University, Zhengzhou, 450052 Henan China

**Keywords:** Cancer therapy, Metastasis, Epithelial–mesenchymal transition, Inflammation, Ferroptosis, Therapy resistance

## Abstract

Iron-dependent lipid peroxidation causes ferroptosis, a form of regulated cell death. Crucial steps in the formation of ferroptosis include the accumulation of ferrous ions (Fe^2+^) and lipid peroxidation, of which are controlled by glutathione peroxidase 4 (GPX4). Its crucial role in stopping the spread of cancer has been shown by numerous studies undertaken in the last ten years. Epithelial–mesenchymal transition (EMT) is the process by which epithelial cells acquire mesenchymal characteristics. EMT is connected to carcinogenesis, invasiveness, metastasis, and therapeutic resistance in cancer. It is controlled by a range of internal and external signals and changes the phenotype from epithelial to mesenchymal like. Studies have shown that mesenchymal cancer cells tend to be more ferroptotic than their epithelial counterparts. Drug-resistant cancer cells are more easily killed by inducers of ferroptosis when they undergo EMT. Therefore, understanding the interaction between ferroptosis and EMT will help identify novel cancer treatment targets. In-depth discussion is given to the regulation of ferroptosis, the potential application of EMT in the treatment of cancer, and the relationships between ferroptosis, EMT, and signaling pathways associated with tumors. Invasion, metastasis, and inflammation in cancer all include ferroptosis and EMT. The goal of this review is to provide suggestions for future research and practical guidance for applying ferroptosis and EMT in clinical practice.

## Introduction

The term “ferroptosis” was originally used in 2012 [1] to refer to a kind of unregulated lipid peroxidation and plasma membrane rupture known as iron-dependent regulatory cell death (RCD) [2]. Ferroptosis can be caused by either intrinsic or extrinsic mechanisms [3]. The extrinsic pathway is activated by the activation of the iron transporters lactotransferrin and serum transferrin or by the suppression of cell membrane transporters such cystine/glutamate transporters (also known as System Xc-). The intrinsic pathway is activated by blocking intracellular antioxidant enzymes such as glutathione peroxidase GPX4 [4]. The peroxisome-ether phospholipid axis also plays an important role in driving ferroptosis. Peroxisomes promote ferroptosis by synthesizing polyunsaturated ether phospholipids (PUFA-ePLs) to provide substrates for lipid peroxidation. Among them, the PUFA chain is the key to ferroptosis sensitization. Cancer cells can downregulate levels of PUFA-ePLs, promoting ferroptosis evasion. This iron-promoting effect occurs not only in tumor cells, but also in normal neurons and cardiomyocytes [5]. Since anti-apoptosis is a hallmark of cancer [6], targeting non-apoptotic RCD pathways may provide a way to stop tumor progression.

The reversible biological process known as EMT allows epithelial cells to briefly change into the quasi-mesenchymal cell state [7–10]. Adhesion junctions and tight junctions created by epithelial cadherin (E-cadherin) molecules on the cell surface are crucial for holding together the apical-basal polar cells that comprise the epithelium in various tissues of the body, thereby maintaining tissue structure [11]. Following EMT activation, E-cadherin expression decreases, resulting in the loss of the characteristic polygonal, pebble-like shape of epithelial cells. The multidirectional EMT-induced transcription factors (EMT-TFs) control EMT by promoting the expression of genes that support mesenchymal cell states in a variety of ways [7–10]. The progression of many different cancer types depends on the activation of EMT in tumor cells [12]. Cancer cells in the context of tumors interact with signaling molecules from tumor-associated reactive stroma to boost the expression of EMT-TFs, which in turn affects the expression of numerous EMT procedure components.

Cancers are aggressive and can spread to other parts of the body, a challenging and often fatal process [13]. Dormant tumor cells of epithelial origin must first migrate and be invasive to travel from the main tumor and enter the bloodstream during cancer metastasis [14]. A portion of the metastatic tumor cells that are still alive will leak out of the blood vessels as circulating tumor cells (CTCs) and begin to move to distant sites where they will colonize [15]. To adapt to their new environment, colonized tumor cells must transition from a quiescent to a proliferative condition. Reduced cancer-related mortality may be achieved by preventing cancer metastasis. Currently, it is believed that the EMT process plays a role in the early phases of the tumor metastatic cascade [16]. In fact, the study of EMT-like modifications in tumor cells has shown that they make the cells more aggressive, and that EMT transcriptome features are closely associated with poor prognoses for various cancers in different patient populations [17].

Inflammation is viewed as a key factor in the growth of cancer and has a substantial impact on this process. Systemic inflammation and local immune responses play a role in the growth of malignant tumors and the survival of cancer patients, according to an increasing number of studies [18]. The connection between chronic inflammation and cancer-related EMT is frequently highlighted in the literature. EMT is frequently induced by inflammation in tumors, and inflammatory mediators (such as soluble factors, oxidative stress, or hypoxia) can encourage the development of EMT-like characteristics in cancer cells while also increasing the production of pro-inflammatory mediators [19].

Ferroptosis and EMT interact, affecting tumor invasion, metastasis, and tumor-related inflammatory response, according to studies. With this knowledge, it may be possible to concentrate cancer treatment efforts on ferroptosis and EMT. Therefore, more research into the alleged pathways is crucial. We explore the processes and functions of ferroptosis and EMT, as well as the crosstalk between them, in the progression of cancer in this review with the aim of positively influencing cancer metastasis as well as cancer-associated inflammation to improve patient outcomes.

## Signaling mechanisms linked to cancer

### Cancer-related signaling pathways in ferroptosis

#### RAS

**Fig. 1:**
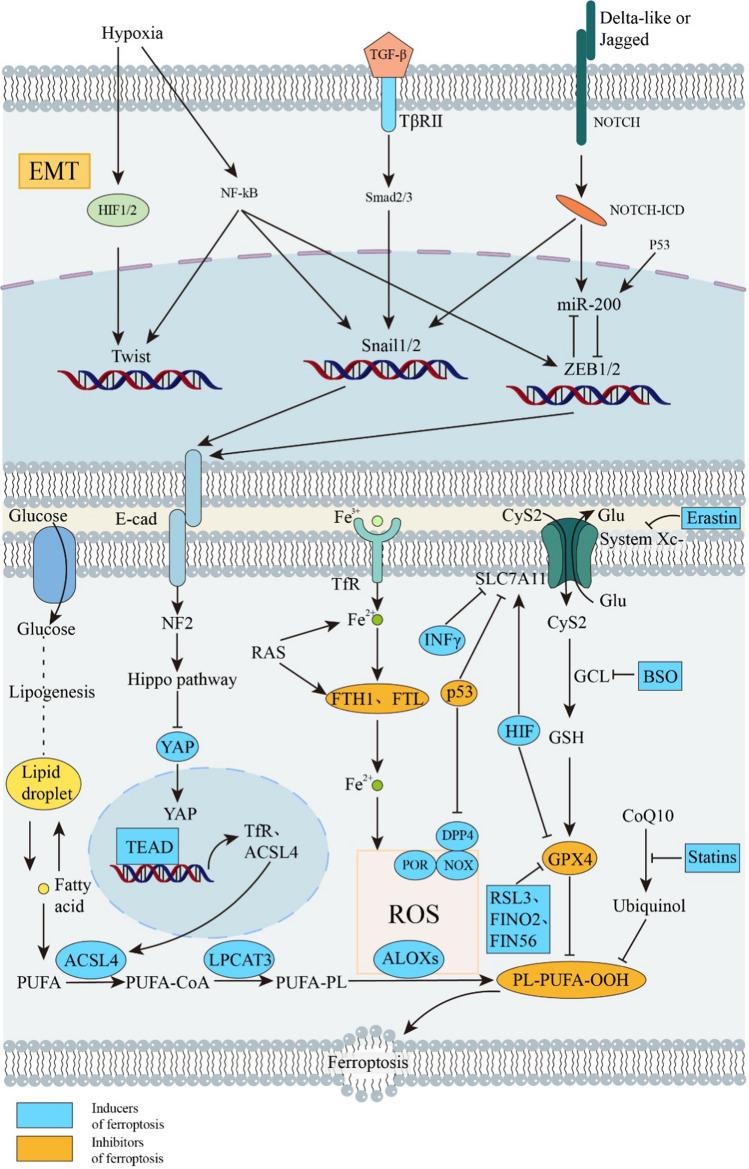
Cancer-related signaling pathways in ferroptosis and EMT. EMT and ferroptosis are associated with each other and tumorigenesis and development through RAS, TP53, hypoxia, TGF-β, and NOTCH-related pathways. Hypoxia, TGF-β, and NOTCH-related pathways affect transcription factors such as HIF, NF-κB, smad, etc., thereby affecting EMT-related transcription factors, including Twist, Snail, ZEB, and thus EMT. P53 also affects intranuclear miR-200 and thus the expression of the transcription factor ZEB. RAS affects ferroptosis by affecting lipid peroxidation by affecting ferritin components (FTH1 and FTL). P53 affects ferroptosis by affecting the Xc-system and lipid peroxidation. Inducers and inhibitors of ferroptosis are described by color in the figure. The blue and orange graphs represent inducers and inhibitors of ferroptosis, respectively. *ZEB* Zinc finger E-box binding, *YAP* yes-associated protein, *TEAD* TEA domain transcription factor, *TfR* transferrin receptor, *FTH1* Ferritin Heavy Chain 1, *FTL* Ferritin Light Chain, *Glu* Glucose, *CyS2* Cystine, *BSO* L-Buthionine-(S,R)-sulfoximine, *RSL3* (1S,3R)-RSL3, *FIN* Ferroptosis inducers, *E-cad* E-cadherin, *Erastin* ACSL4, acyl-CoA synthetase long chain family member 4, *ALOXs* lipoxygenases, *CoA* coenzyme A, *CoQ10* coenzyme Q10, *DPP4* dipeptidyl peptidase 4, *FA* fatty acid, *G6PD* glucose-6-phosphate dehydrogenase, *GCL* glutamate-cysteine ligase, *GPX4* glutathione peroxidase 4, *GSH* glutathione, *LPCAT3* lysophosphatidylcholine acyltransferase 3, *NOXs* NADPH oxidases, *PL* phospholipid, *PLOOH* phospholipid hydroperoxides, *POR* cytochrome p450 oxidoreductase

RAS family oncogenes (HRAS, NRAS, and KRAS) are the most often mutated in cancer, yet RAS mutant tumors have been challenging to treat [20]. The RAS protein is considered to as “undruggable” since it is so hard to discover inhibitors. Erastin and Ras-selective lethal3 (RSL3) are inducers of ferroptosis that are selectively lethal to oncogenic RAS mutant cell lines, collectively referred to as RAS selective fatal (RSL) chemicals [21]. Only tumor cells with the RAS mutation undergo ferroptosis in response to a ferroptosis inducer (bearing oncogenic Ras). The expression of transferrin receptor protein 1 (TfR1) increased whereas that of ferritin (FTL and FTH1) decreased in ferroptosis-sensitive cells with a RAS mutation (Fig. 1) [22]. Due to increased iron intake and decreased iron storage, iron accumulates more during ferroptosis. Blocking the Ras/Raf/MEK/ERK pathway may reduce Erastin-induced ferroptosis in RAS mutant cancer cells [23]. An innovative ferroptosis mutagen is the natural substance β-elemene, which is effective against human colorectal cancer (CRC) cells with KRAS mutation and inhibits tumor growth by inducing ferroptosis. Combining β-elemene with cetuximab could potentially provide a therapeutic alternative for CRC patients with RAS mutation [24].

#### TP53

The TP53 tumor suppressor gene is the most commonly mutated gene in cancers, and its wild-type form can inhibit tumor growth through various mechanisms [25]. Wild-type p53 slows ferroptosis in human and animal cancer cells by inducing the expression of the downstream target CDKN1A (encoding p21CIP1/WAF1) and inducing secondary cystine deficiency [26]. The TP53 mutation and subsequent p53 inactivation, however, allow tumor cells to survive and proliferate rapidly [25]. A transcription factor called p53 has the capacity to trigger apoptosis and ferroptosis [27]. P53 promotes ferroptosis by preventing SLC7A11 transcription in cancer cells (Fig. 1). The acetylation-deficient mutant p53 3KR (K117R, K161R, and K162R) was unable to induce apoptosis in lung cancer cell lines but fully preserved the ability to induce ferroptosis [28]. p53 R273H and R175H can nevertheless limit the creation of SLC7A11 by blocking the actions of other transcription factors [29], despite the fact that they cannot bind to DNA. Certain metabolism-related genes, such as SAT1, FDXR88, and GLS2, are direct targets for p53-mediated ferroptosis under a variety of conditions [4].

The dipeptidyl peptidase DPP4 and p53 interact directly, and this interaction may help p53 prevent NOX-mediated lipid peroxidation in CRC cells (Fig. 1). Additionally, by boosting CDKN1A expression in fibrosarcoma cells, p53 may reduce ferroptosis [30]. Through non-transcriptional inhibition of DPP4 activity, TP53 prevents erastin-induced ferroptosis. The lack of TP53 enhances plasma membrane-associated DPP4-dependent lipid peroxidation, which results in ferroptosis by preventing intranuclear aggregation of DPP4 (Fig. 1). These findings demonstrate a role for TP53 and DPP4 in regulating lipid metabolism and have the potential to offer a therapeutic approach by generating ferroptosis for the treatment of colorectal cancer [31]. LncRNA P53RRA encourages cell cycle arrest, death, and ferroptosis; ferroptosis is characterized by intracellular iron and lipid reactive oxygen (ROS) concentrations. We discovered a correlation between the levels of these two ferroptosis markers and P53RRA. By interacting with G3BP1, P53RRA suppresses the ferroptosis gene in a p53-dependent way. P53RRA elevates p21 and MDM2 levels. P53RRA reduced the expression of numerous metabolic genes, including SCL7A11, which regulates iron content and is involved in ferroptosis. By keeping p53 in place, P53RRA encourages ferroptosis in the nucleus [32]. Without the aid of p53, the two proteins MDM2 and MDMX, which bind to p53 and regulate its stability, induce ferroptosis in cancer cells [33].

#### Hypoxia-inducible factor (HIF)

Solid tumors are characterized by hypoxia, which encourages cell growth, survival, and metastasis and is resistant to chemotherapy and radiotherapy [34]. HIF, the main regulator of hypoxia, is made up of an oxygen unstable-subunit (including HIF1, EPAS1(HIF2α, and HIF3α) and an expression-related subunit (ARNT) [35]. The HIF-1 signaling pathway is found through the analysis of 259 genes linked with ferroptosis and their potential roles. It has the highest enrichment in KEGG, suggesting that it is tied to ferroptosis [36]. Ferroptosis can be controlled in two different ways by HIF, according to research [37].

HIF is the main mechanism that induces cell ferroptosis in renal clear-cell carcinoma. When the ferroptosis inhibitor is added after inhibiting GPX4 in renal clear-cell carcinoma, cell death is decreased because HIF inhibits GPX4 to cause ferroptosis. HIF-1α and HIF-2α mediate the sensitivity of clear-cell carcinoma to ferroptosis. The hypoxia-induced lipid drop-associated protein (HILPDA), which activates the HIF-2α pathway, makes cells more susceptible to ferroptosis. HIF-2α selectively enriches polyunsaturated lipids through the activation of HILPDA, and when GPX4 is lost or consumed, this accumulation of polyunsaturated lipids (PUFA)-phospholipid peroxide results in cell ferroptosis. Given its poor prognosis and extensive treatment resistance, clear-cell carcinoma may now have a therapeutic target [38].

HIF-1α, the main transcription factor regulating hypoxia, also influences angiogenesis and cancer through altering the activation of hypoxia-related genes [39, 40]. For example, in gastric cancer cells, HIF-1α has been shown to inhibit ferroptosis by increasing the expression of SLC7A11 at the post-transcriptional level and stabilizing SLC7A11 mRNA at the transcriptional level (Fig. 1). The ability of gastric cancer cells to withstand ferroptosis induced by Erastin or RSL3 may depend on the nucleoplasmic distribution ratio of ELAVL1 [41]. According to other studies, the activation of HIF-2 upregulates the lipid and iron regulatory genes in colorectal cancer cells and mouse colon tumors, making the cells prone to ferroptosis. Second, by oxidizing cysteine irreversibly, HIF-2α activation encourages the production of ROS and ferroptosis. When HIF-2α is brought down, ROS and antioxidant cells are less likely to perish in vivo and in vitro. The results show that colorectal cancer can be treated by exploiting the susceptibility of HIF-2α-dependent cancer cells [42].

### The cancerous EMT-related signaling pathway

#### TGF-β

Members of the TGFβ family include three TGFβs, two activins, numerous bone morphogenetic protein (BMP) homologs, as well as different homodimers and heterodimers of ligands. All of these ligands work by activating a pair of transmembrane bispecific kinase receptors [43]. The majority of malignancies have elevated TGFβ1 expression and activation, which encourages epithelial plasticity and can accelerate EMT, a requirement for cancer cell invasion and metastasis [7, 10, 44]. The TGFβ pathway triggers EMT in a variety of unique ways. Together with EMT-inducing transcription factors (EMT-TFs) such as SNAIL, SLUG, ZEB1, and TWIST, TGF-β-induced SMAD complex transcription activates the mesenchymal genes for waveform protein and fibronectin. E-cadherin is inhibited by this (Fig. 1) [45]. The expression of TGF ligands can be increased if EMT-TFs are activated, resulting in a positive feedback loop through autocrine signaling that helps cells sustain the expression of EMT programs. This demonstrates the presence of reciprocal signals between TGF-β and EMT-TFs pathway users. TGFβ can control the expression of EMT-TFs by inducing post-translational modifications. TGFβ triggers EMT via regulating long non-coding RNAs and microRNAs (lncRNAs). The miR-200 microRNA family hinders the synthesis of the ZEB1 protein, which in turn prevents the transcription of miR-200, creating a double negative feedback loop (Fig. 1). TGF-β reduces miR-200’s bioavailability, which in turn stimulates ZEB1-mediated EMT [43, 46]. TGF-β has been shown to contribute to EMT in a number of malignancies. A range of ECM cell types is stimulated to upregulate and activate TGF-β in response to both short-term and long-term liver injury [47]. Activated TGF-β promotes both the activation of hematopoietic stem cells and the EMT-induced fibroblast to mesenchymal transition. TGF-β has a strong inhibitory effect on the proliferation of healthy melanocytes. EMT, proliferation, metastasis, and immunological tolerance in malignancies are all stimulated by TGF-β. The chemotherapy regimens ginsenoside Rb2 [48] and tanshinone II A [49] have therapeutic benefits for CRC [47, 50] by inhibiting TGF-induced EMT and angiogenesis, respectively. Pancreatic adenocarcinoma (PDA) frequently harbors KRAS oncogenic mutations, which greatly enhance TGF-β’s capacity to trigger EMT. SMADs expressing RREB1 directly control the expression of mesoderm genes and EMT transcription factors in pluripotent progenitor cells, as well as the expression of EMT transcription factors and fibrous factors in cancer cells. The universality of the TGF-SMAD- RREB1 mechanism also offers the door for a fuller understanding of the role of TGF-β in organ fibrosis and cancer pathogenesis and provides a consistent framework for examining EMT throughout the course of development and regeneration [51]. Global chromatin modifications, histone variations (H2A. Z), and novel chromatin modifiers (e.g., UTX, Rad21, PRMT5, RbBP5, etc.) are thought to be crucial for the control of EMT transcription factors (EMT-TFs) and EMT markers (EMT-MS) in TGF-β-induced EMT, according to epigenetics [52].

#### Notch

The Notch signaling pathway was first identified to be active in acute T-lymphocytic leukemia/lymphoma (T-ALL/T-LL), and aberrant Notch signaling pathways have also been discovered in a number of solid tumors [53, 54]. Components of the NOTCH route are heavily expressed near the boundaries of neoplastic invasion and commonly show EMT markers such as vimentin, indicating that this pathway is important for the regulation of EMT [12, 55–57]. Through the finger structure, which functions as a transcriptional blocker, Snail1 and Snail2 (Slug) are connected to the E-box primitives (5’-CANNTG-3’) in the target initiators (for instance, the E-cadherin gene (CDH1) promoter). Notch is considered to activate EMT through controlling the transcription of a number of EMT-TFs, including SNAIL and SLUG (Fig. 1) [58]. Lowering E-cadherin expression, which also sparks the production of fibronectin and vimentin, is the initial stage of EMT [59]. When the Notch intracellular domain (Notch-ICD) is overexpressed, E-cadherin [60] is lost and snail expression increases. Notch signaling directly contributes to the induction of EMT [58, 61] via Snail (Fig. 1).

In a model with carcinogenic Kras expression and Notch1 deletion of lung adenocarcinoma, the study showed that the function of Notch1 is to prevent p53-mediated apoptosis by modulating the stability of p53, which leads to tumorigenesis [58, 62]. The Notch signaling pathway promotes EMT by enlisting HIF-1α and HIF-2α and can also increase the expression of Snail through a complex framework [63]. The interplay of the Notch-miR-200-ZEB1 ring controls EMT and transfer capacity (Fig. 1) [58]. TGF- mediates the engagement of SMAD with snail promoters and causes the onset of EMT in NSCLC through ligand-receptor binding [64, 65].

### Crosstalk between ferroptosis and EMT

Numerous studies have shown that the pathway related to ferroptosis and the pathway related to EMT share many junctions and interactions. The main contributing aspect is the considerable interaction between ferroptosis and EMT that occurs as the tumor develops.

#### Ferroptosis increases EMT inhibition

2,2’-Dipyridone hydrazone dithiocarbamic acid (DpdtC), which likewise suppresses EMT by producing ROS generated from iron, generates high quantities of ROS (Fenton reaction). A DpdtC homologue named DpdtbA suppresses EMT in a way that is comparable to DpdtC. 2,2’-Dipyridyl ketone hydrazone dithiocarbamic acid s-butyric acid (DpdtbA), a ferroptosis, exhibits remarkable anticancer effects against gastric and esophageal cancer cells [66]. Both in normoxic and hypoxic conditions, DpdtbA prevents EMT. Mechanistic studies have shown a relationship between PHD2/HIF-1α activation and the inhibition of EMT. DpdtbA also promotes the growth of ferritin phages, and the oxygen and ferrous ions generated by the Fenton reaction may contribute to the activation of PHD2 and the p53 response [67]. This demonstrates that DpdtbA suppresses EMT by activating the p53 and PHD2/HIF-1α pathways [66].

Additionally, it has been discovered that iron chelators lessen lung epithelial cell EMT, mitochondrial malfunction, and cell death brought on by cigarette smoke exposure (CSE). This might be a new area to focus on while treating pulmonary fibrosis [68].

#### The occurrence of EMT leads to greater sensitivity to ferroptosis

Upregulation of Bach1 is associated with EMT in glioma cells. More significantly, proteomic study showed that Bach1’s principal method of glioma invasion promotion depends heavily on the extracellular matrix (ECM). Bach1 has a two-way impact on gliomas. The important glioma invasion regulator Bach1 has been found to coordinate a number of ECM-related activities. Bach1 overexpression also lowered the requirement for glioma ferroptosis induction [69]. Drugs that induce ferroptosis may enable the transition from Bach1 high expression induced tumor invasion to tumor suppression in gliomas [70].

Gambogic acid (GNA), a flavonoid found in garcinia, is cytotoxic to melanoma cells because they are so aggressive [71]. TGF-β1 induces EMT in melanoma cells in addition to increasing lipid peroxidation levels and making cells more vulnerable to ferroptosis. GNA prevents GPX4 from being produced via inducing p53, which results in defects in ferroptosis and intracellular lipid peroxidative repair [72].

Ectopic MIB1, E3 ubiquitin ligase-expressing A549 cells go through EMT and are propelled to migrate along the Notch-dependent route. The breakdown of NRF2, which weakens cell antioxidant defenses and raises sensitivity to inducers of ferroptosis, is facilitated by MIB1 in a Notch-independent way. However, ferroptosis resistance and NRF2 accumulation ensue after MIB1 deletion. All things considered, these results suggest that MIB1 may work to weaken the main antioxidant transcription factor NRF2 to influence ferroptosis positively [73].

The researchers found that E-cadherin-mediated cell-to-cell contact reduces ferroptosis by triggering the Hippo signaling pathway, which in turn makes the ferroptosis-causing transcriptional co-regulator YAP less active. YAP is activated when EMT begins, increasing vulnerability to ferroptosis [74].

Inducing HNC cells to take on a mesenchymal phenotype, which promotes ferroptosis, and epigenetic reprogramming of EMT, which aids in enhancing ferroptosis sensitivity in HNC cells, are two potential combination therapies for the treatment of ferroptosis-resistant malignancies [75].

HDAC inhibitor therapy can make SW13 cells more susceptible to ferroptosis by inducing EMT and altering intracellular iron levels. These findings are crucial since a variety of other cell types have also been shown to exhibit an interstitial phenotype after being treated with HDAC inhibitors [76].

GPX4 inhibits ferroptosis in cancer cells, including EMT, that are resistant to therapy. Cells lacking the EMT marker CDH1 are more vulnerable to stimuli that trigger ferroptosis because CDH1 negatively regulates ferroptosis [77]. These findings provide a therapeutic alternative for osimertinib-resistant NSCLC cells that undergo EMT. Treatment strategies for NSCLC cells that are resistant to osimertinib include inhibiting the NF-kB pathway or focusing on GPX4 to stop EMT [78].

#### The role of iron and ferritin in EMT and ferroptosis

One way cancer cells take up iron is by binding iron to ferritin and entering cells through transferrin receptors (TFR1) [79]. Ferritinophagy is a type of cell-selective autophagy that causes ferritin-bound iron to be released to become free iron. Mitochondrial ferritin protects cells from reactive oxygen species, thus preventing ferroptosis. In fibroblasts and cancer cells, ferritinophagy promotes ferroptosis through NCOA4-mediated ferritinophagy degradation and subsequent release of labile iron. NCOA4-mediated ferritinophagy also increases the susceptibility of glioblastoma cells to ferroptosis [80, 81]. In addition, studies have found that ferritin does not provide labile iron through NCOA4-mediated ferritinophagy, but through reductive mobilization [82].

**Fig. 2:**
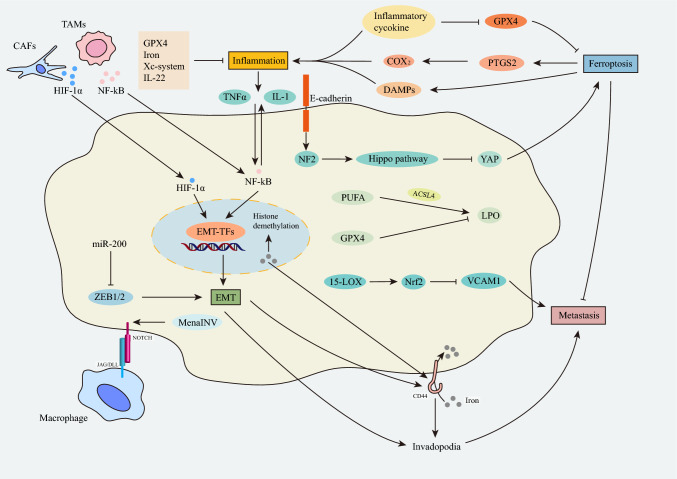
Crosstalk between ferroptosis and EMT in tumor inflammation and tumor metastasis. Genetic mutations in the E-cadherin-NF2-Hippo-YAP pathway sensitize cancer cells to ferroptosis and drive metastasis. GPX4 and 15-LOX inhibit tumor metastasis. Ferroptosis can directly increase the expression of PTGS2 of COX2, increase the secretion of inflammatory signals, and promote the occurrence of inflammation. Conversely, inflammation can also affect the occurrence of ferroptosis through the secretion of inflammatory factors. Inflammatory factors such as TNF-α and IL-1 produced by inflammation, as well as HIF-1α and NF-κB produced by TAMs and CAFs, can indirectly affect the transcription factor expression of EMT. *CAFs* cancer-associated fibroblasts, *TAMs* Tumor-associated macrophages, *LPO* Lipid Peroxide, *15-LOX* 15-lipoxygenase, *MenaINV* Mena invasive, *PTGS2* Prostaglandin-Endoperoxide Synthase 2, *VCAM1* Vascular Cell Adhesion Molecule 1, *Nrf2* NF-E2-related factor 2, *DAMPs* damage associated molecular patterns

In recent years, another mechanism of iron uptake has been discovered in mesenchymal tumor cells. CD44 mediates cellular uptake of specific metals, including iron and copper [83]. Cells undergoing EMT tend to absorb iron in the CD44-mediated pathway, and CD44 is upregulated due to epigenetic changes related to specific demethylation of repressive marks at the gene locus, which are controlled by iron-dependent demethylases. This upregulation enables mesenchymal cells to absorb iron to a higher degree [84]. Studies have shown that methylation status is a key component of epithelial–mesenchymal plasticity, while demethylases regulate EMT and its inverse counterpart [85]. Nuclear iron acts as a metal catalyst for histone demethylation and as a rate-limiting factor for epigenetic plasticity, promoting selective oxidative demethylation of key DNA or histone residues on chromatin to dynamically control epithelial–mesenchymal states [86, 87] (Fig. 2). Ferritin is involved in the development of cancer through EMT, and ferritin expression is upregulated in breast cancer cells with a mesenchymal phenotype, accompanied by an increase in the level of nuclear H-ferritin and a decrease in the level of labile iron [88]. However, ferritin has also been reported to have the opposite effect on cancer proliferation. This may be tissue-specific or cancer-specific effects [82].

## The function of ferroptosis and EMT in the development of tumors

### Invasion and metastasis activation

#### Ferroptosis in the invasion and metastasis of cancer

Genes in the E-cadherin-NF2-hippo-YAP pathway typically have malignant mutations that encourage metastasis, prevent cancer cells from dying, and boost their resistance to conventional cancer treatments (Fig. 2). The fact that these changes make cancer cells sensitive to ferroptosis makes them both attractive indicators for iron-induced therapies and a suggestion for novel therapies that cause ferroptosis [74]. The interaction of ferroptosis with lipid metabolism has also been connected to tumor invasion and metastasis. First of all, studies have shown that the main regulator of ferroptosis, GPX4, inhibits invasion and spread of tumors. Increased metastatic activity in 27HCR cells is causally connected to higher GPX4 pathway activity (or reduced stress). GPX4 deletion reduces the ability of 27HCR and B16F10 melanoma cells to metastasis in comparison to melanoma cells of the wild type. Since it demonstrates how vital it is to treat metastatic cancer by targeting the GPX4 axis, this discovery has significant therapeutic significance [89]. Additionally, both GPX4 and 15-LOX have the ability to block VCAM1, which aids in the promotion of tumor spread and angiogenesis. This could be achieved by upregulating endogenous heme oxygenase-1 by activating nuclear factor erythroid 2-related factor 2 (Nrf2) (Fig. 2) [90, 91]. Second, ferroptosis requires an accumulation of iron, and both excessive ferroportin expression and the iron chelator Dp44mT can stop tumor growth. Third, ACSL4 and 15-LOX are key regulators of lipid metabolism and LPO production during ferroptosis, increasing the invasion and migration of breast and prostate cancer cells [91–93].

#### EMT in cancer invasion and metastasis

EMT is associated with the loss of epithelial markers and cell–cell connections as well as an enhanced expression of mesenchymal markers [94], and it is seen in the invasive front of the tumor. Nearby to the connective tissue proliferative stromal is this area of the tumor. A high level of miR-200 (an inhibitor of ZEB1 and ZEB2) was found to be a reliable predictor of successfully metastatic breast cancer in a study in a mouse model and in human tissue. Indicating that decreased ZEB1/ZEB2 expression was linked to reduced metastasis, and higher miR-200 family expression was highly associated with metastatic-free survival (Fig. 2) [46, 95–99]. With chemotherapy and miR-200-mediated EMT blocking, recent advancements in miRNA-based therapeutics show significant promise for stopping metastasis in breast cancer models. Early adjuvant therapy that blocks EMT may make cancers more sensitive to cytotoxic drugs and immunotherapies [100]. Some examples of EMT-TFs necessary for tumor metastasis include Twist1, Snail1, and Prrx1. For example, increased SNAIL1 expression resulted in E-cadherin-mediated intercellular adhesion loss, significant morphological transformations from epithelial to mesenchymal spindle cells, and an uptick in invasive and metastatic behaviors in breast cancer cells. Twist1 promotes invadopodia, which leads to stromal degradation, and EMT by making Snail2 decrease E-cadherin (Fig. 2). TWIST1 has been found to be an important downstream target of HIF-1α in head and neck cell lines [101]. The tumor microenvironment also affects the tumor invasion and metastasis. The macrophage-cancer-endothelial-endothelial tumor metastatic microenvironment (TMEM), a three-cell complex, promotes the transendothelial migration of tumor cells. Physical contact between macrophages and tumor cells results in the formation of ivadopodia [102–104] via the Notch1/MenaINV signaling pathway in tumor cells (Fig. 2). In a different study, breast tumor-active cells could travel to distant organs through hematogenous metastasis when they had the TGF-Smad2/3 EMT activation signal, but cells without this signaling pathway were more likely to passively migrate in groups to lymph nodes [105].

### Inflammation

#### The connection between cancer's ferroptosis and inflammation

Ferroptosis has a dual function in inflammation, acting as both a pro- and an anti-inflammatory. How can ferroptosis, therefore, result in inflammation? The generation of eicosanoids, AA metabolism, and ferroptosis are closely connected. According to studies, ferroptosis can increase the expression of COX2 and PTGS2, hasten AA metabolism, and promote the release of inflammatory signaling molecules (Fig. 2) [106]. According to research, GPX4 dramatically lowers the quantity of cellular lipid hydroperoxides, which is a contributing element to the onset of inflammation [107]. Numerous side-soluble cancer cell types can release high migration swarm 1 (HMGB1) in an autophagy-dependent manner [108, 109]. HMGB1 strongly contributes to the pathophysiology of inflammation as an injury-related molecular pattern (DAMP) that increases inflammation (Fig. 2) [110, 111]. Numerous inflammatory cytokines, such as TNF, PGE2, IL-1, IL-6, and IL-1, have been reported to have a direct effect on the levels and activity of GPX4 in cancer cells [112]. For instance, TNF treatment of cells resulted in GPX4 downregulation that is permanent and may also lead to ferroptosis [109].

What role does ferroptosis play in the anti-inflammatory effects? Because ceramides are necessary for the skin’s barrier function, human dermatosis linked to ceramide deficit and ceramide synthesis knockout mice is characterized by skin inflammation [113, 114]. According to studies, solenopsin, a ceramide analog, greatly improves skin when used to treat inflammatory skin conditions. Other improvements include elevated IL-22, a cytokine raised in both psoriasis and cutaneous squamous cell carcinoma, as well as downregulated GPX family members, such as GPX4, and improved iron input in cells [115]. Each of these proteins has an association with ferroptosis [116]. Ceramides and solenopsin may physiologically cooperate to increase reactive oxygen species and decrease selenoproteins like GPXs. In light of this, the researchers hypothesized that ferroptosis might be a physiological mechanism that is therapeutic and can protect the skin against inflammatory and cancerous conditions [117].

#### The relationship between EMT and inflammation in cancer

Inflammatory compounds including TGFβ, tumor necrosis factor (TNF), interleukin-1 (IL-1), IL-6, and IL-8 stimulate transcription factors like Smads, NF-κB, STAT3, Snail, Twist, and ZEB to promote EMT (Fig. 2). TGF-β is thought to activate EMT [118]. TNF-α stimulates Snail1 promoter activity, EMT, and protein stabilization in cancer cells. When paired with other inflammatory agents, continuous TNF-α exposure induces tumor cells to multiply and grow through angiogenesis, which can lead to EMT in human cancer cell lines, CXCR2 and CXCR3 expression in renal cell cancer cell lines, and IFN-γ and TNF-α expression in thyroid cancer [119]. As a result of IL-1β, additional pro-inflammatory mediators might be released at higher amounts [120].

The activation of NF-κB appears to be a crucial participant in both pathways, and TNF-α and hypoxia both contribute to the production of EMT in malignancies through a number of transcription factor expression pathways [121]. Greater production of the typical pro-inflammatory cytokines tumor necrosis factor (TNF-α) and IL-1 is required for increased NF-κB activation and the recruitment of inflammatory cells to obstructed kidneys (IL-1). Active NF-κB creates a cycle that maintains inflammatory signaling by controlling the production of pro-inflammatory cytokines (Fig. 2). These cytokines attract inflammatory cells, and tumor cells gather once more at the tumor-stroma interface with TAMs and CAFs to produce NF-κB and hypoxia-inducible factor (HIF-1α), creating a microenvironment that can encourage tumor growth (Fig. 2) [121]. HIF-1 efficiently promotes EMT in malignancies like kidney carcinoma [122]. NF-κB can directly promote the expression of several potent EMT inducers, including the Snail1 and ZEB factors (Fig. 2). The relationship between inflammation and EMT appears to be a feature of cancer growth, therefore, it may be considered [120] to employ specific anti-EMT treatments in combination with anti-inflammatory drugs to treat cancer.

## Ferroptosis and EMT in cancer therapy

### Potential ferroptosis-inducing cancer therapies

#### Systematic therapy

The formation of tumors, activation of systemic Xc-transporters, increased GSH metabolism and GPX4 activity, inhibition of lipid peroxidation, and iron metabolism are all effects of ferroptosis inhibition in cancer [123]. Ferroptosis can be promoted using system Xc-inhibitors, GPX4 inhibitors, GSH synthesis inhibitors, and lipid peroxidation stimulants, which will stop tumor growth (Table 1).

**Table 1:** Selected drugs associated with ferroptosis in oncology

Target	Drug	Tumor type	Effect	References
Xc-system	Sulfasalazine	Lymphomas, pancreatic cancer, and lung cancer	Induce	[153, 154]
	Sorafenib	Hepatocellular and renal cell carcinoma and thyroid cancer	Induce	[155]
	Erastin	Cholesterol-reducing reagents	Induce	[156]
	TMZ	Gliomas	Inhibit	[157, 158]
GPX4	β-elemene(Cetuximab)	Colorectal cancer (KRAS-mutant)	Induce	[159]
	Withaferin A	Breast cancer, osteosarcoma, neuroblastoma treatment	Induce	[134]
	Altretamine	Lymphoma, sarcoma, ovarian cancer treatment	Induce	[160]
GSH	Cisplatin	Ovarian cancer, pancreatic cancer, urothelial cancer, lung cancer	Induce	[161, 162]
	BSO	Melanoma, neuroblastoma	Induce	[163, 164]
Fe	Artesunate	Pancreatic cancer, Burkitt’s Lymphoma	Induce	[165–167]
	Ironomycin	Pancreatic cancer	Induce	[168]
	Neratinib	Breast cancer, CRC	Induce	[167]
	Lapatinib	Breast cancer	Induce	[167]
	Salinomycin	Various solid tumor types	induce	[87]
CoQ10	Statins	Cholesterol-reducing reagents	Induce	[116]

*GPX4* Glutathione Peroxidase 4, *TMZ* Temozolomide, *GSH* glutathione, *BSO* Buthionine sulfoximine, *CRC* colorectalcancer

The cystine/glutamate anti-transporter (Xc-system), which is made up of the light chain subunit SLC7A11 and the heavy chain subunit SLC3A2, serves as the major transporter. As a result of its absence from normal physiology and the high level of expression it displays in cancer, SLC7A11 is a promising candidate for use as a cancer therapeutic target. This is because targeting SLC7A11 may allow for the selective killing of tumor cells and the impediment of tumor growth while sparing normal cells or tissues. The current work highlights two strategies for SLC7A11, one of which focuses on preventing SLC7A11-induced dependency on glucose or glutamine and the other on directly inhibiting SLC7A11-mediated cystine absorption. To start, lower the activity of this enzyme directly using SLC7A11 cystine transporter inhibitors such as erastin, IKE, sulfadiazine, sorafenib, and HG106 [124, 125]. These medications stop SLC7A11 from absorbing cystine, which results in lipid peroxidation and spherocytosis. Second, T alters the dependence on glucose in SLC7A11 high cancer cells by limiting the uptake of glucose with GLUT inhibitors. Reduced glucose availability in SLC7A11 high tumor cells leads to disulfide stress, which accelerates cell death [126]. Similar to this, glutaminase inhibitors like CB-839 were administered to cells with high SLC7A11 expression to induce glutaminase dependence [127]. Glutaminase inhibition reduces intercellular degeneration caused by glutamine in cancer cells with high levels of SLC7A11. Research suggests that malignancies with certain oncogenic alterations, such as KRAS mutant tumors, depend on cystine absorption mediated by SLC7A11, perhaps rendering them more sensitive to SLC7A11 inhibitors [128–130]. Furthermore, P53 has been shown to inhibit SLC7A11 transcription and result in ferroptosis in liver cancer [131].

GPX4 is a promising target that can reduce complex lipid peroxides and ferroptosis-kill clinically resistant cancer cells. Due to RSL3’s attachment to and inactivation of GPX4, ferroptosis is brought on by an accumulation of ROS. RSL3 has been shown to have a powerful fatal effect on tumorigenic RAS cell lines and to inhibit the development of fibrosarcoma in mice models. By inactivating GPX4, the small molecule atratamine also causes ferroptosis. CoQ10 and SectRNA are blocked by FIN56, which also accelerates the disintegration of GPX4 and results in ferroptosis [132]. FIN56 also stimulates squalene synthase, an enzyme involved in the production of cholesterol. FINO2 directly oxidizes ferrous iron while indirectly inhibiting GPX4-induced ferroptosis [133]. Withaferin A, a naturally occurring ferroptosis inducer, inhibits the growth of xenoblastoma and dose-dependently inactivates GPX4 in neuroblastoma [134]. The antimalarial medication artemisinin derivative dihydroartemisinin has a high bioavailability and triggers ferroptosis by GPX4 inhibition and cysteine deprivation [135].

Increased glutathione levels in cells shield them from oxidative harm. Ferroptosis is initiated once GSH production is compromised. BSO reduces tumor burden in mice and improves the susceptibility of melanoma and neuroblastoma cells to chemotherapy by inhibiting glutathione synthesis rate-limiting enzyme (GCL), inactivating GPX4, and inducing cell death [136, 137]. Similar to this, the growth of prostate and breast cancer xenograft tumors is inhibited and mice with chronic lymphocytic leukemia survive longer when treated with cyst enzymes, a substance that improves GSH depletion efficiency and hence decreases GPX4 activity [137]. Additionally, it increases mouse survival in chronic lymphocytic leukemia models.

Raises the amount of cellular ROS and initiates lipid peroxidation, which furthers ferroptosis. Ferroptosis is thought to be prevented by the lipophilic antioxidant CoQ10, also known as ubiquitin, which snares free radicals [138]. Two different forms of statins, simvastatin and corner vastatin, can result in ferroptosis by reducing GPX4 biosynthesis and obstructing the production of CoQ10. Afrin A also induces ferroptosis in neuroblastoma by increasing lipid peroxidation via heme oxygenase 1-mediated heme degradation [134].

Siderophosis is a physiologically mediated iron-dependent Fenton response. Raising the level of Fe^2+^ in the cell iron pool is, therefore, a useful treatment for cancer [139]. Since the development of nanotechnology, iron-based nanoparticles have been employed in studies on cancer treatment. By releasing iron (Fe^2+^) or iron (Fe^3+^) ions in acidic lysosomes, iron-based nanomaterials induce ferroptosis in tumor cells and lipid peroxidation at tumor sites [140].

#### Immunotherapy

Several immunosuppressive cells, including M2 macrophages, Treg cells, and MDSCs, prevent ferroptosis and maintain cell activation by expressing large quantities of GPX4 or other components. If ferroptosis is activated, the capacity of these cells to produce malignancies may be inhibited [141]. The ferroptosis inducer ZVI-NP increases the anti-tumor effect by polarizing M2 macrophages to M1, decreasing the frequency of Treg, decreasing the expression of PD-L1 on tumor cells and PD-1/CTLA4 on CD8 + T cells, and increasing the anti-tumor immune response [141]. A lack of the GPX4 gene can lead to lipid peroxide (LPOs) overaccumulation and ferroptosis, which boost IL-1β production and enhance T helper 17 (Th17) cells’ anti-tumor immune response [142]. Polymorphonuclear myeloid-derived suppressor cells (PMN-MDSCs) can transfer lipids to DC cells, prevent the cross-presentation of DC cells, and rely on myeloperoxidase for lipid peroxidation, all of which contribute to their tumor-promoting activity [143].

Immune cells also carry out anti-tumor immunological function by releasing cytokines that promote the ferroptosis activity of tumor cells. As an illustration, IFN produced by CTLs inhibits the expression of the Xc-system, activates the STAT1 pathway, and increases the amount of iron that accumulates in cells, causing ferroptosis. Comparatively, transforming growth factor-1 (TGF-β1) produced by macrophages can promote ferroptosis by inhibiting the Xc-system’s transcription through SMAD signaling.

In some situations, ferroptosis may also have the effect of promoting tumor growth. Pancreatic cancer cells may release KRAS-G12D by binding to advanced glycation end-product-specific receptors (AGER) during ferroptosis. By causing the release of arginine (ARG), IL-10, and TGF-β, this may cause the M1 phenotype to polarize into the M2 phenotype, induce adaptive immunosuppression, and eventually encourage tumor growth [137, 144].

### Three main strategies for targeted-EMT therapy

Targeted EMT therapy today uses three primary strategies [145]. First, cancer is prevented by obstructing upstream signaling pathways, such as ligand neutralizing antibodies, dummy receptors, or inhibitors that obstruct TGF-β, NF-κB, EGFR, cMET, WNT, and Notch signaling [146, 147]. Several pro-inflammatory signals, such as TNF-α, are also potent inducers of EMT [148]. The EMT driver molecule is the second one. EMT-TFs are the main controllers and forces behind the EMT process. The second therapy strategy either targets mesenchymal cells and ECM or inhibits MET. By limiting cell plasticity, mesenchymal-specific proteins prevent tumor cells from developing resistance to standard treatments and encourage re- or trans- differentiation. A third therapeutic strategy focuses on the relationship between cancer cells and the ECM. Integrins are crucial in mediating the interaction of cancer cells with the extracellular matrix (ECM), activating signals that are crucial for the formation, expansion, and metastasis of solid tumors [147, 149]. Integrins are a key component of tumor growth and metastasis, making them an attractive target for cancer therapy. The breakdown of the ECM and the EMT program are both triggered by the synthesis of MMP family proteases, which makes it simpler for cancer cells to circulate and invade distant tissues [150]. Therefore, MMP inhibitors may be suggested as potential cancer therapies. Targeted EMT therapy has the potential to stop tumor cells from invading and migrating inside the primary tumor, but this approach is only effective if the cancer cells have not yet disseminated widely. Finding the ideal timing to begin treatment is crucial because EMT develops; otherwise, anti-EMT therapy can have the opposite impact of what was intended. The opposite pathway may be promoted during anti-EMT therapy, leading to the expansion of MET and promoting the colonization and metastasis of circulating tumor cells [151]. Restriction of EMT activities may also affect the body’s capacity to heal itself because EMT is essential for the physiological reaction to trauma and wound healing [152]. Because of this, EMT has two sides to its focused therapy, focusing on both the effects of its beneficial and unfavorable effects [145].

## Conclusions and perspectives

Ferroptosis and EMT are the two primary mechanisms that drive cancer growth and both rely on multiple signaling pathways. Mesenchymal cancer cells, which exhibit a more fibroblast-like morphology, are generally more susceptible to ferroptosis than cancer cells with epithelial characteristics. EMT increases cells’ vulnerability to ferroptosis and drives their transition from epithelial to mesenchymal phenotypes. This transition leads to an increase in lipid peroxide generation and the synthesis and metabolism of polyunsaturated fatty acids. The resulting peroxidation of PUFAs further sensitizes cancer cells to ferroptosis by suppressing GPX4, xCT, GSH, and anti-ferroptosis regulator proteins through ROS/RNS increases. Ferroptosis also suppresses EMT, suggesting that targeting both mechanisms in tumor cells may be a promising anti-cancer strategy. However, further research is needed to fully understand the underlying mechanisms connecting ferroptosis and EMT, including the development of specialized drugs that selectively induce ferroptosis in cancer cells. Additionally, identifying biomarkers that can help identify and monitor ferroptosis and EMT in patients will be a significant area of focus for future research. Overall, this review highlights the emerging connections between ferroptosis, EMT, and tumor progression, and the potential of targeting these mechanisms for novel anti-cancer therapies.
